# Gene Expression in Porcine Bulbourethral Glands

**DOI:** 10.3390/ani14071115

**Published:** 2024-04-05

**Authors:** Victoria Noto, Barbara Jean Nitta-Oda, Trish Berger

**Affiliations:** Department of Animal Science, University of California, Davis, CA 95616, USA

**Keywords:** androgen receptor, five alpha reductase, allometric growth, *GPER*, boar

## Abstract

**Simple Summary:**

Bulbourethral glands are a major accessory sex gland in the boar. Their rate of growth is similar to general body growth at 6 weeks of age, but subsequently, bulbourethral glands grow faster than the general body. This study evaluated the expression of two genes that would increase androgen signaling despite the relatively low androgen concentrations during this interval. Neither gene exhibited increased expression to provide an androgen-mediated stimulus for growth. Hence, the normally occurring reduction in endogenous estrogen synthesis, which would alleviate G protein-coupled estrogen receptor 1 (GPER)-mediated inhibition of growth, may be the cause for the initial stimulus of growth during this late juvenile interval.

**Abstract:**

The porcine bulbourethral glands produce a gel-type secretion. Although the role of these contributions to reproductive success remains murky, the bulbourethral glands are major accessory sex glands in this species. Isometric growth in the early neonatal interval is followed by allometric growth in the late juvenile interval (6 to 11 weeks of age), while circulating endogenous steroids are low. The rate of allometric growth increases during the peripuberal interval (16 to 20 weeks of age) when systemic testosterone is relatively high. Gene expression for androgen receptor (*AR*) and for the steroid 5 alpha-reductase 2 (*SRD5A2*) enzyme that synthesizes the more potent androgen dihydrotestosterone from its precursor was evaluated by qPCR analyses of bulbourethral gland tissue. Tissues were collected from control boars (2 weeks to 40 weeks of age) and from littermates of these boars treated with letrozole to suppress endogenous estrogen synthesis. Gene expression for these two key proteins in androgen signaling was quite low during the initial allometric growth in the late juvenile and prepuberal intervals, suggesting that this initial growth was not primarily stimulated by androgens. These observations are consistent with a more direct estrogen-mediated inhibition of growth via GPER previously proposed, with the sensitivity extending into the late juvenile interval when estrogens as well as androgens are normally relatively low.

## 1. Introduction

A more complete understanding of reproductive physiology may provide opportunities to improve animal production by advancing reproductive management. Growth and development of the male reproductive tract have been extensively studied in the mouse animal model, but growth, development, regulation, and function of the bulbourethral glands, which are particularly prominent in the boar, have only been minimally studied [1,2]. The diversion of energy to maintain such a large mass of bulbourethral glands suggests a positive rather than neutral contribution of these glands in the boar, presumably more than lubrication. Gel particles containing zinc-binding proteins could function to swab the ejaculatory pathway [3]. The small amount of gel in the initial fraction of semen might cleanse this path prior to the ejaculation of the sperm-rich fraction. The large amount of gel at the end of ejaculation might help remove the microbe-friendly seminal plasma to reduce microbial colonization.

Previous observations indicated that boars experiencing reduced endogenous estrogens beginning in the early juvenile interval had smaller accessory sex glands postpuberally [4]. However, boars experiencing reduced endogenous estrogens seemed to have larger bulbourethral glands prepuberally. In contrast, peripuberal growth of the bulbourethral glands in the boar may be stimulated by estrogens [5]. Deviation from isometric growth of the accessory sex glands in general, including bulbourethral glands, is believed to result from androgens acting through the androgen receptor (AR). This suggested earlier maturation of accessory sex glands in the presence of reduced endogenous estrogens and that the endogenous estrogens might affect the AR, as suggested by Prins and colleagues for the prostates of estrogenized neonatal rats [6]. Hence, this study focused on local gene expression of five alpha reductase type 2 (*SRD5A2*), the enzyme that converts testosterone to the more potent androgen, dihydrotestosterone (DHT), and *AR* in porcine bulbourethral glands during postnatal development. As bulbourethral gland tissue differentiates from the same embryonic origin as prostatic tissue [7], increased growth of porcine bulbourethral glands during the early juvenile interval during suppression of endogenous estrogen synthesis might be independent of the hypothesized alteration in androgen signaling [8]. Reduced inhibition of growth by reduced estrogen signaling through G-protein-coupled estrogen receptor 1 (GPER) might cause the observed allometric growth. To evaluate the response of bulbourethral glands to endogenous estrogens during the juvenile interval, littermates to control boars were treated with the aromatase inhibitor letrozole. Since aromatase is the enzyme that synthesizes estradiol, inhibition of aromatase reduces estradiol synthesis in the testis and, as a result, reduces serum estradiol.

## 2. Materials and Methods

### 2.1. Animals and Treatments

Boars (n = 28; 14 littermate pairs) between the ages of 2 and 40 weeks were from established lines developed from Durocs, Hampshires, Yorkshires, and Pietrains by PIC USA (Sygen International, Franklin, KY, USA) and housed at the UC Davis Swine Center. Animals were housed in indoor farrowing pens until weaning between 3 and 4 weeks of age, continued in indoor nursery pens, subsequently moved to finishing pens, and individually housed in boar pens after 20 weeks of age without regrouping beyond the initial pre-weaning group. Post-weaning diets were a corn:soybean meal-based diet supplemented with vitamin and mineral premixes and formulated for age-related dietary needs.

**Figure 1:**
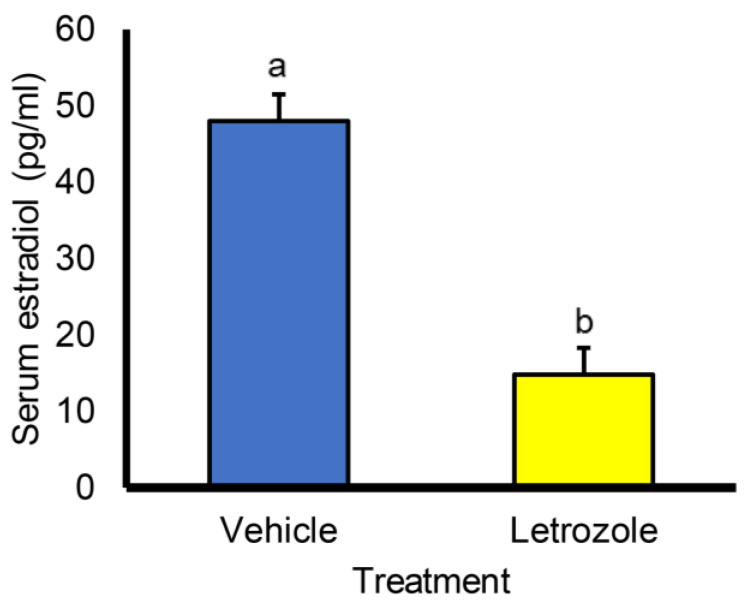
Serum estradiol is rapidly reduced following letrozole treatment. Values represent means ± SEM from 26 littermate pairs of 2-week old boars one week after treatment with 0.1 mg letrozole/kg body weight. Data redrawn from animals and treatments previously described [9,10,11,13]. a, b *p* < 0.0001.

Bulbourethral glands were collected from littermate pairs of boars at the following postnatal ages: early juvenile 2 weeks (n = 2 treatment, n = 2 control), juvenile 6.5 weeks (n = 4 treatment, n = 4 control), early puberal 16 weeks (n = 6 treatment, n = 6 control), and postpuberal 40 weeks (n = 2 treatment, n = 2 control) [9,10,11,12]. Ages were chosen to represent physiological states and apparent transition points in control animals. One individual from each littermate pair had been randomly assigned to weekly oral treatment with 0.1 mg of letrozole [4-4′-(1H-1,2,3-triazol-1-yl-methylene)-bis-benzonitrile] (Ciba-Geigy/Novartis, Basel, Switzerland)/kg body weight, and the remaining littermate received canola oil. Weekly dosing began at 1 week of age and continued through 6 weeks of age, except for samples obtained at 16 weeks of age and for those samples obtained at 2 weeks of age. Boars providing tissues at 16 weeks of age received weekly dosing from 11 to 16 weeks of age. Letrozole treatment rapidly reduces circulating estrogen levels [4], as illustrated in Figure 1. Treatment and results from other analyses of all of these animals, including steroid levels, were previously described and indicated that letrozole treatment dramatically reduced estrogen synthesis as expected [9,10,11,12].

### 2.2. Tissue Collection and Analyses

At tissue collection, bulbourethral glands were individually weighed, and a piece of bulbourethral gland was flash frozen and stored at −80 °C prior to processing. Tissue was subsequently disrupted with glass lysis beads in a BioSpec Mini-Beadbeater-24 (BioSpec Products, Inc., Bartlesville, OK, USA) during homogenization in QIAzol Lysis Reagent^®^ (QIAGEN, Germantown, MD, USA). The RNA was isolated via chloroform extraction and 2-propanol precipitation. The cDNA was synthesized using a Revertaid kit (Thermo Scientific, Waltham, MA, USA). Relative gene expression was analyzed by qPCR using a QuantStudio 3 qPCR machine (Applied Biosystems, Waltham, MA, USA). Arginyl-tRNA synthetase *(RARS2*) was used as the reference gene, and ΔC_t_ was calculated for each sample from the means of triplicate wells. The primers for *RARS2* and *SRD5A2* expression were previously described [13,14].

Data were subjected to analysis of variance (ANOVA) using R Statistical Programs 4.1.1 [15] and a mixed effects model (lmer function; [16]). Litter was considered a random effect, and treatment or age were fixed effects.

## 3. Results

**Figure 2:**
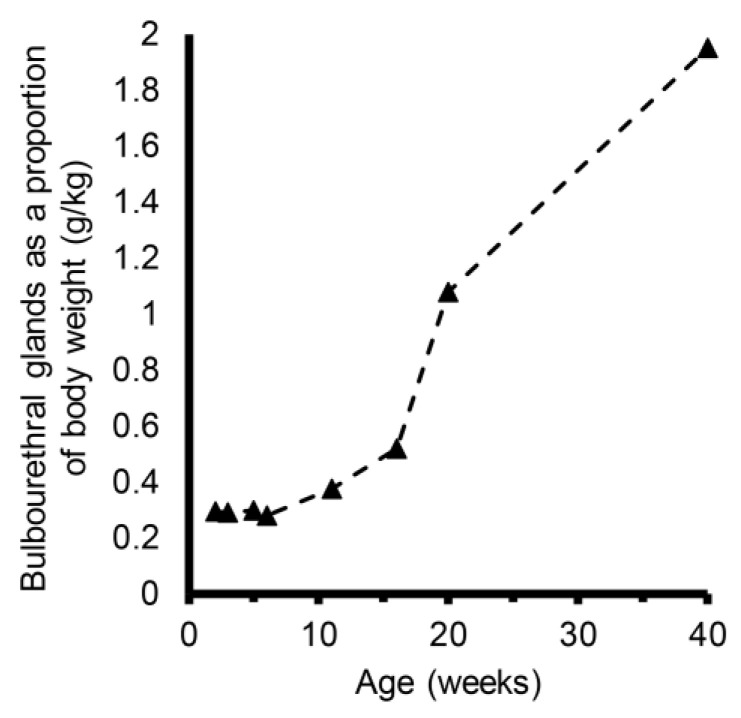
Bulbourethral glands as a proportion of body weight in control boars. Black triangles represent mean proportional weights (g/kg) from 3 to 6 boars at each age and dashed line illustrates profile. Gland weights appeared to increase at a faster rate in the peripuberal interval (16–20 weeks) than earlier or later although allometric growth between 6 and 11 weeks of age appeared similar to the rate of allometric growth between 11 and 16 weeks of age.

**Figure 3:**
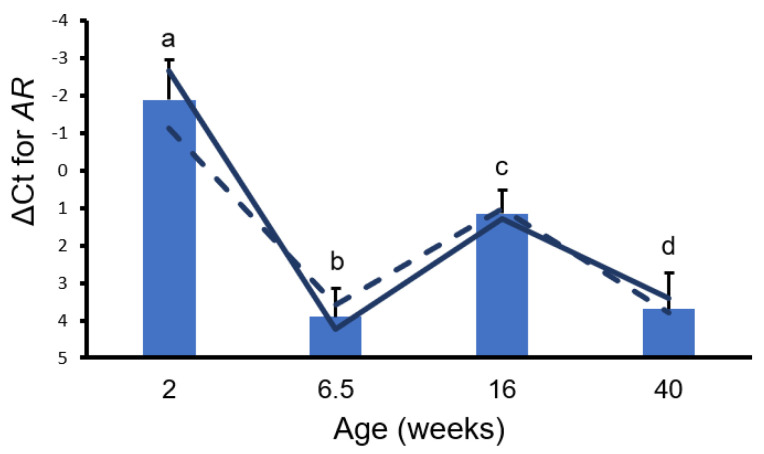
Delta Ct Values for *AR*. Bars represent means from control and treated boars at each age with SEM from analysis. a, b *p* < 0.001. a, c; c, d *p* < 0.05. b, c; a, d *p* < 0.01. Solid lines represent values only from control boars and dashed lines represent values from littermates treated with letrozole (low estrogen).

**Figure 4:**
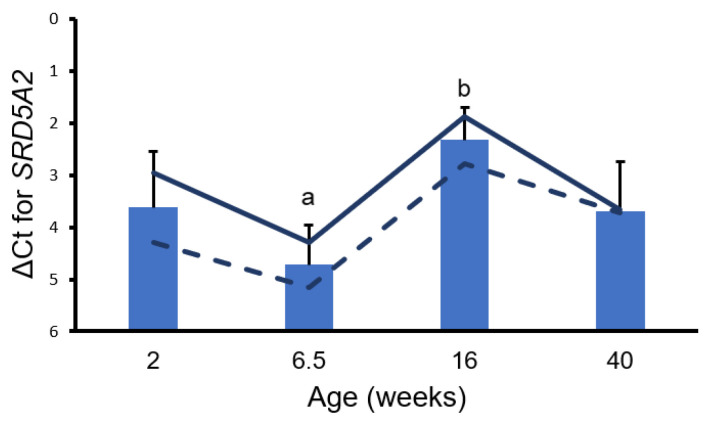
Delta Ct values for *SRD5A2*. Bars represent means from control and treated boars at each age with SEM from analysis; a, b *p* < 0.05. Solid lines represent values only from control boars and dashed lines represent values from littermates treated with letrozole (low estrogen).

The growth of bulbourethral glands appeared isometric through 6 weeks of age (Figure 2). Allometric growth of bulbourethral glands was observed to begin at 6 weeks of age, with a much greater rate of allometric growth observed between 16 and 20 weeks of age. Changes were observed in both *AR* and *SRD5A2* gene expression in porcine bulbourethral gland tissue with age. *AR* expression decreased between 2 weeks and 6.5 weeks of age (*p* < 0.001) and subsequently increased to 16 weeks, but not to neonatal levels, before falling again (Figure 3). Expression of *SRD5A2* appeared to decrease between 2 and 6.5 weeks of age and subsequently increased peripuberally (16 weeks of age) (Figure 4). Expression of *AR* and *SRD5A2* was similar in boars with normal estrogen concentrations and those with suppressed endogenous estrogens.

## 4. Discussion

The similar developmental origins of the bulbourethral glands and the prostate suggest that gene expression related to steroid signaling might have similar temporal profiles. Similar expression profiles would provide support for bulbourethral gland responses, including growth, to be used as partial corroboration of the more anatomically entrenched porcine prostate in experimental protocols. The temporal pattern of *AR* expression in bulbourethral glands relative to physiological timepoints resembled the temporal expression profile observed in the porcine prostate [13]. Similar to what was previously observed in the prostate, reduced endogenous estrogens had no effect on *AR* expression in porcine bulbourethral glands; this contrasts with the response of the rat prostate to pharmacological estrogens [6]. However, the temporal profile of *SRD5A2* expression in the porcine bulbourethral glands does not resemble the *SRD5A2* expression profile in the prostate, as expression of *SRD5A2* was at its lowest level in the bulbourethral glands at the juvenile timepoint (6.5 weeks of age) but high at the same timepoint in the prostate [13]. Differences in the *SRD5A2* temporal expression profile indicate these two organs, both derived from the urogenital sinus, have clear differences in gene expression profiles. Secretion from the porcine bulbourethral glands is obvious prenatally [17]; this is earlier than visible secretion from the prostate or seminal vesicles, indicating initial maturation differs temporally among the organs. This work demonstrates that the *AR* was not affected by the letrozole treatment, and the resulting reduction in signaling from endogenous estrogens; previous work indicated testosterone was not affected by the letrozole treatment which reduced endogenous estrogens [18]. However, letrozole treatment does stimulate increased growth of the bulbourethral glands as well as the prostate and seminal vesicles [8]. Hence, the stimulation of growth by reduced estrogens was apparently not due to altered androgen signaling. Additionally, no morphological effects on murine bulbourethral glands by altered steroid levels have been detected prenatally [19].

At least two alternatives to androgen-mediated stimulation of allometric growth have been suggested. In humans, T cells may stimulate the proliferation of cells in the bulbourethral glands [20]. The possibility exists that T cells might be involved in the initial allometric growth in control boars, although further investigation is beyond the scope of the current work. Alternatively, the normally occurring reduction in circulating estrogens may remove estrogen signaling through *GPER*, which inhibits growth. This explanation is consistent with the stimulation of growth during letrozole-induced reduction in estrogens and the absence of a growth response to fulvestrant treatment, suggesting a GPER-mediated effect in the early juvenile interval [8].

## 5. Conclusions

Although a shift to allometric growth was observed beginning at approximately 6 weeks of age during an interval of low systemic testosterone and estradiol, these data on gene expression indicate the expression of *AR* and *SRD5A2* is unaffected by endogenous estrogens. Hence, increased sensitivity to androgen signaling does not explain the bulbourethral glands’ shift to allometric growth. The initial observed increase in growth rate at 6 weeks of age is consistent with a reduction in GPER-mediated inhibition of growth due to the normal reduction in estrogens.

## Data Availability

Data are presented within the text of this report and in the previously published, referenced papers.
